# Bacterial maze runners reveal hidden diversity in chemotactic performance

**DOI:** 10.15698/mic2019.08.688

**Published:** 2019-07-01

**Authors:** M. Mehdi Salek, Francesco Carrara, Vicente Fernandez, Roman Stocker

**Affiliations:** 1 Institute for Environmental Engineering, Department of Civil, Environmental and Geomatic Engineering, ETH Zurich, 8093 Zurich, Switzerland.

**Keywords:** bacterial chemotaxis, microfluidics, T-maze geometry, phenotypic heterogeneity, Escherichia coli, chemotactic sensitivity

## Abstract

Chemotaxis allows microorganisms to exploit gradients in chemical stimuli to find nutrient resources and hosts or escape noxious substances. Thus, the life of individual microbes in their natural environments is a continual sequence of decisions based on the perceived chemical gradients. However, it has remained unclear to what extent the chemotaxis properties vary among cells of one species, and whether there is a spectrum of different ‘decision makers' within populations of bacteria. In our recent study (Salek, Carrara *et al.*, Nature Communications 10 (1), 1877), we combine microfluidic experiments with mathematical modeling to demonstrate that even in clonal populations, bacteria are individuals with different abilities to climb chemical gradients.

Scientists have generally considered the chemotactic ability of bacteria to be a bulk characteristic of a species or a population, where average values suffice to describe their movements. However, it is known that cells are subject to intracellular biochemical noise, which can significantly affect their performance. Such phenotypic heterogeneity, or non-genetic diversity, arises in bacterial populations from stochastic gene expression and partitioning of proteins, functional molecules, and mRNA at cell division, even in the absence of genetic variation or environmental cues. Chemotaxis, a fundamental behavioral characteristic of many species of bacteria, is governed by multiple proteins involved in the chemotactic pathway. Differential expression of these proteins has the potential to generate heterogeneity in chemotactic performance among cells in navigating the environment.

Bacteria swim by rotating their flagella using molecular motors. Characteristic patterns of motility have been described for a wide range of species, including microbes living in soil, the human gut, and the ocean. For example, *Escherichia coli* swims using a run and tumble motion. Cells swim straight during a run by bundling together multiple rotating flagella, and reorient in a random direction during a tumble. In this way, bacteria perform a random walk, allowing them to effectively explore new territories. Cells perform chemotaxis by biasing their swimming preferentially toward higher concentrations of attractants. They do so by reducing the probability of tumbling when moving toward a source of chemoattractants. Due to biochemical noise, individual cells may have different baseline levels of swimming speed or tumbling rate, or vary in their sensitivity to detect chemical sources. For example, if a cell on average performs fewer tumbles per unit of time, it spends more time in running and thus will travel a greater distance; mathematically, this is equivalent to stating that the cell increases its diffusivity by reducing its tumble bias. Another cell might be more efficient in tracking a weak chemical gradient; in other words, it has an increased sensitivity in the chemotactic pathway due to a higher pathway gain that amplifies the perceived signal. There are thus multiple phenotypic traits, including, but not limited to, the tumble bias and the pathway gain, that contribute to the chemotactic velocity of a cell, which is the average drift of the cell in the direction of a chemical gradient.

In order to quantify this heterogeneity in phenotypic traits involved in the chemotaxis pathway and its contribution to the navigation performance of bacterial cells, we designed a microfluidic T-maze in which cells navigate chemical gradients, so that the better chemotactic decision-makers in a population are spatially sorted from the weaker chemotaxers **(Figure 1)**. The use of microfluidic technology is revolutionizing our ability to interrogate individual bacterial cells. By combining the use of hydrogels to generate steady-state chemical gradients with classical microfluidics to fabricate microscale features and live-image single cells, we were able to precisely control the microscale environment and monitor bacterial behavior. This allowed us to study microbes using similar concepts to those used by scientists to study animals. Inspired by binary mazes – classical tools in ecological studies used to assess the preference of macro-organisms for different cues – we designed an iterative microfluidic T-maze to observe single cells navigating in the presence of a chemical gradient. The microscale T-maze is designed in such a way that each bacterium is faced with a series of sequential decisions when encountering a chemical gradient at the consecutive junctions of the maze. The chemoattractant gradient is constant throughout; however, the mean concentration level increases in subsequent junctions. Unlike previous one-dimensional chemotaxis assays, the two-dimensional branching design of our microscale T-maze is advantageous to quantify the concentration of bacteria through consecutive junctions as they swim through the maze.

**Figure 1:**
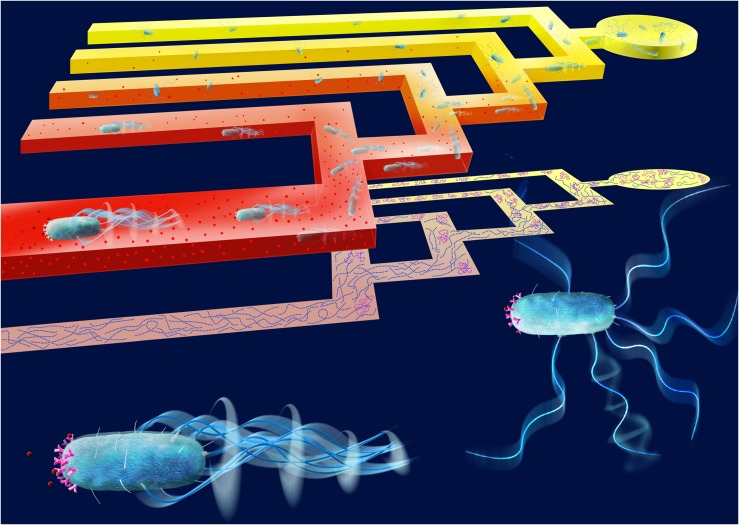
FIGURE 1: Behavioral experiments with bacteria swimming through a microfluidic T-maze reveal strong heterogeneity in chemotactic sensitivity. A hydrogel-ased microfluidic T-maze creates a steady chemical gradient (red molecules) by eliminating flow in the swimming channel. As cells swim from right to left through the maze, they face a series of decisions at successive T-junctions, with the choice of approaching or avoiding the chemical stimulus (top). The T-maze can thus sort the better chemotaxers within a monoclonal population of bacteria or can be used to sort among populations with different behavioral characteristics (as shown in the schematic projection of individual bacterial trajectories, center, showing cells with strong (blue) and weak (purple) chemotactic performance). Also shown are cells in the two stages of motion underlying chemotaxis: a cell in running mode with high chemotactic sensitivity (left), bearing a higher number of chemoreceptors, and a cell in the tumbling mode (right), with lower sensitivity to the chemical gradient. Image credit: Salek, M.M., Carrara, F. Gorick, G. and Stocker, R.

By coupling microfluidic experiments in a branching geometry with mathematical modeling, we could demonstrate the existence of heterogeneity in both tumble bias and pathway gain in a monoclonal population of *E. coli*. Using single-cell video microscopy, we measured the relative number of cells in the up- and down-gradient portions of each junction. The comparison of the behavior of cells reaching the up-gradient portion of subsequent junctions showed that the cells were being sorted according to variations in chemotactic sensitivity. Our mathematical model of chemotaxis that accounted for various potential sources of phenotypic heterogeneity revealed that, as cells navigate through the maze, they are sorted for both lower tumble bias and higher pathway gain at subsequent junctions. Most notably, the cells with low tumble bias, which diffuse more efficiently, reach the more advanced junctions at earlier times; however, the cells with high pathway gain are sorted more stably over time. The two-dimensional branching design of our microscale T-maze offers several advantages over previous one-dimensional chemotaxis assays, yielding a stable sorting of cells according to their chemotactic performance, and providing a means to quantify the concentration of bacteria through consecutive junctions, and thus isolate the contribution of different sources of phenotypic heterogeneity to variation in chemotactic performance.

The demonstration that chemotactic sensitivity is highly heterogeneous within a bacterial population opens the door to a broad set of new questions in microbial ecology, on the functional role of such heterogeneity in bacterial motility. Rather than being a bulk characteristic of a population, as it has been largely treated to date, chemotactic sensitivity can be highly variable among cells within a population, and this variability may serve specific functions, including the ability to optimally balance exploration and exploitation in patchy and time-varying environments. This may provide an evolutionary advantage for the bacteria, for example, in the context of migratory bet-hedging strategies, and thus represent an adaptive trait.

Non-genetic diversity in bacterial traits has long been known in the biomedical sciences; for example, it is thought to play a role in antibiotic resistance. More recently, environmental studies including our work have shown that this diversity affects fundamental behaviors of bacteria, such as locomotion and chemotaxis. This work represents a potential source of cross fertilization between environmental and medical sciences, where such intrapopulation heterogeneity in the response to gradients promises to inspire new lines of research in domains such as immunology, *in vitro* fertilization, cancer and drug resistance. In all these fields, tools such as our microfluidic T-maze to sort cells and to elucidate the mechanisms that result in a spectrum of functionalities within a cell population exposed to a chemical landscape will be important in advancing our ability to harness the potential advantages or limit the disadvantages that bacterial heterogeneity brings.

